# The Effect of Different Particle Size Distribution on the Quality of Rice Flour

**DOI:** 10.3390/foods15020204

**Published:** 2026-01-07

**Authors:** Qinghua Yue, Xiya Song, Yuxia Yang, Jingwen Qin, Yue Li, Xunda Wang, Jiangtao Lin

**Affiliations:** College of Food Science and Engineering, Henan University of Technology, Zhengzhou 450001, Chinayangyuxia1999@163.com (Y.Y.); qinjingwen02@163.com (J.Q.); 15729397701@163.com (Y.L.); 202191007@stu.haut.edu.cn (X.W.)

**Keywords:** rice flour, particle size, composition, physicochemical property

## Abstract

Rice flour, as an essential food ingredient, exhibits processing and end-use properties that are critically influenced by its particle size distribution (D10, D50, D90). This study systematically investigates the effect of varying particle size fractions on the chemical composition, color, water absorption, pasting behavior, thermal properties, and rheological characteristics of rice flour. Our results indicate no statistically significant differences in the major chemical constituents across different particle size ranges (*p* > 0.05). However, finer particles demonstrated increased whiteness (89.94 to 90.52) and higher levels of damaged starch. A consistent decline was observed in several pivotal parameters—including peak viscosity, final viscosity, breakdown, setback, onset temperature, peak temperature, conclusion temperature, gelatinization enthalpy (ΔH), storage modulus (G′), and loss modulus (G″)—with decreasing particle size, although only marginal variations were noted for the finest D and E fractions. Notably, the coarsest fraction exhibited the highest gelatinization enthalpy (ΔH = 11.12 J/g). These findings elucidate the fundamental role of particle size distribution in modulating the multifunctional properties of rice flour, providing a theoretical foundation for its targeted application and quality optimization in food industry practices.

## 1. Introduction

As a cornerstone of global food security, rice stands among the world’s most vital cereal crops, ranking third in total production after wheat and maize, yet serving as the principal dietary staple for over half of the human population [1]. China plays a pivotal role in the worldwide rice supply chain, possessing the second-largest cultivation area after India while consistently leading in total output. According to national statistics, Chinese rice production reached 209.61 million tons in 2019, rising to 211.86 and 212.85 million tons in 2020 and 2021, respectively, before moderating to 208.50 million tons in 2022 [2]. Beyond direct consumption, rice undergoes extensive processing into diverse derived products, including extruded items such as instant rice noodles and rice vermicelli, prepared foods like instant rice, congee, rice cakes, and traditional delicacies including zongzi, tangyuan, and ciba, baked goods such as rice pots and rice bread [3], expanded products exemplified by rice cakes and puffed snacks [4], as well as fermented specialties including rice wine, vinegar, and related beverages [5]. Most of these convenience-oriented products rely on rice flour as their primary intermediate material, whose functional properties profoundly influence the quality, stability, and consumer acceptance of the final output [6]. Hence, a systematic understanding of rice flour characteristics is indispensable for advancing value-added rice processing technologies.

Milling represents a critical unit operation in rice refinement, wherein grains are mechanically comminuted into flour with targeted granulometric properties. This process predominantly modifies the physical architecture of rice kernels while largely preserving their intrinsic chemical composition. Common milling strategies encompass wet, dry, and semi-dry approaches [7], each exerting distinct effects on flour composition, physicochemical functionality, particle size distribution, and starch damage extent—factors that collectively govern gelatinization behavior and ultimately shape end-product quality [6]. Wet milling generally yields flours with narrower particle size distributions and minimal starch damage, resulting in superior performance in subsequent processing; however, this approach entails extended operational duration, higher equipment demands, and environmentally challenging wastewater output [8]. In contrast, dry milling offers operational simplicity and cost efficiency, though it frequently produces flour with elevated levels of damaged starch, which can adversely affect sensory and visual characteristics of finished products [9,10]. Emerging evidence suggests that semi-dry milling represents a promising intermediate pathway, delivering flour quality parameters situated between those obtained by dry and wet milling [11,12].

Flour quality is predominantly governed by particle size distribution and starch damage extent, both widely regarded as pivotal indicators of processing suitability and end-product performance [13,14]. Reduced particle size correlates strongly with increased starch damage, diminished gelatinization temperatures and enthalpy, enhanced hydrophobic characteristics, and improved oil-binding capacity—attributes linked to smoother noodle surfaces and modified textural properties in rice cakes [15,16,17]. Nevertheless, systematic investigations examining the influence of particle size on other critical flour attributes—including color evolution, hydration behavior, and thermal properties—remain comparatively limited, constraining the rational design of flour for specific applications.

Therefore, this study employs early indica rice as raw material, comminuting grains to achieve five distinct flour fractions characterized by controlled particle size distributions, wherein 90% of particles pass through designated sieve apertures. Through comprehensive characterization of these fractions, we elucidate the effects of granulometric properties on rice flour quality, thereby establishing a theoretical framework to deepen the study on the physical and chemical properties of rice flour with different particle sizes and promote its better application in rice products.

## 2. Materials and Methods

### 2.1. Materials

The indica rice used in this work was obtained from Changde, Hunan, China. Analytical-grade chemicals, namely potassium iodide, sodium thiosulfate, and boric acid, were purchased from Kemiou Chemical Reagent Co., Ltd. (Tianjin, China). These characteristics directly determine the final quality of rice noodles. A high amylose content—amylose being the linear component of starch with strong intermolecular interactions—contributes to high gel strength. During the processes of gelatinization (heating) and retrogradation (cooling), the robust gel structure formed by amylose enables rice noodles to maintain their integrity during subsequent cooking, reducing the likelihood of breaking or becoming overly soft and mushy [18].

### 2.2. Rice Flour Preparation

Rice flour was prepared using a semi-dry milling method. The selected rice had a moisture content between 14% and 15%. It was then dried to approximately 12% moisture content before being milled into flour. A mill (Model: MLU-202, Bühler, Uzwil, Switzerland) was used, with the roller gap set at 100 µm, the fast roller speed at 500 rpm, and a speed ratio of 2.5:1. The milled flour was subsequently sieved through meshes of 186, 156, 124, 108, and 90 μm to obtain different particle size fractions.

Prior to milling, the rice grains were conditioned to a target moisture content. A precise amount of deionized water was added to the rice, which was then sealed in a plastic bag and allowed to equilibrate for 5 h to achieve uniform water absorption. The conditioned rice was then dried at 40 °C until the moisture content reached approximately 12%. Finally, the rice was ground and classified into five distinct particle size groups: coarse (A, 156–186 μm), medium (B, 124–156 μm; C, 124–108 μm), and fine (D, 108–90 μm; E, <90 μm).

The amount of tempering water was calculated according to the following equation:

Added deionized water (g) = M × (W^1^ − W^0^)/(1 − W^1^), where M is the initial mass of the rice (g), W^1^ is the target moisture content after tempering (%), and W^0^ is the initial moisture content of the rice (%).

### 2.3. Chemical Compositions

The total starch content was quantified using the Total Starch Assay Kit (K-TSTA, Megazyme International Ltd., Wicklow, Ireland). Similarly, the amylose content was determined by the corresponding Amylose Assay Kit (Megazyme International Ltd., Wicklow, Ireland). The protein, moisture, lipid, and ash contents were assessed according to the official AACC Methods 46-11A, 44-19, 30-10, and 08-01, respectively. The damaged starch content in the rice flour was measured with a Damaged Starch Analyzer (SDmatic, Chopin Technologies, Paris, France).

### 2.4. Particle Size Distributions

Particle size distributions of the rice flour fractions were analyzed using a dry dispersion particle size analyzer (NKT6100, Shandong Naikete Analytical Instrument Co., Ltd., Jinan, China) following dry dispersion principles. In this method, air was used as the dispersion medium, and the refractive index for the rice flour particles was configured at 1.55.

### 2.5. Color Properties

The color parameters (L*, a*, b*) of the rice flour samples were determined with a colorimeter (CR-410, Konica Minolta, Tokyo, Japan). The instrument was calibrated with a standard white calibration plate prior to measurement. The Hunter whiteness value was subsequently derived from these parameters based on a previously established method [19], as defined by Equation:

Hunter whiteness = 100 − [(100 − L*)^2^ + (a*)^2^ + (b*)^2^]^1/2^, where L* denotes lightness on a scale from 0 (black) to 100 (white); a* represents the red-green coordinate (positive values indicate redness); and b* represents the yellow-blue coordinate (positive values indicate yellowness).

### 2.6. Water Absorption, Water Solubility, Swelling Power

To determine the hydration properties, the classical method was adopted with some modifications [20]. Hydration Properties were measured at different temperatures. Specifically, 0.1 g of rice flour was thoroughly homogenized in 20 mL of distilled water, after which the dispersion was centrifuged at 3000× *g* for 30 min. The resulting supernatant was transferred to a tared aluminum dish and evaporated to constant weight in an oven at 105 °C. The water absorption index was measured by agitating the suspension in a water bath at 90 °C for 30 min. The water absorption index (WAI), water solubility index (WSI), and swelling power (SP) were calculated based on the following equations: WAI (g/g) = Wet sediment weight/Sample dry weight, WS (%) = Dry supernatant weight/Sample dry weight × 100, SP (g/g) = Wet sediment weight/(Sample dry weight × (1 − WS)/100).

### 2.7. Scanning Electron Microscopy

The microstructure of rice flour particles was examined using a scanning electron microscope (SEM, Apreo 2, Thermo Fisher Scientific, Waltham, MA, USA). The samples were mounted on aluminum stubs and sputter-coated with a thin layer of gold prior to imaging to enhance conductivity [21].

### 2.8. Pasting Properties

The pasting properties followed the previous methods with a Rapid Visco Analyzer (RVA-TecMaster, Perten Instruments, Stockholm, Sweden) [22]. First, a suspension was prepared by weighing 3.0 g of rice flour (on a 12% moisture basis) into an RVA canister with 25 mL of distilled water. Subsequently, the pasting cycle was executed as follows: hold at 50 °C for 1 min, heat to 95 °C in 3 min 45 s, maintain at 95 °C for 2 min 30 s, cool to 50 °C in 3 min 51 s, and hold at 50 °C for 1 min 24 s. Standard parameters such as peak viscosity (PV), trough viscosity (TV), final viscosity (FV), breakdown (BD), and setback (SB) were obtained from the instrument’s software (RVA, 2025).

### 2.9. Thermal Properties Measurement

We measured the thermal properties using a Differential Scanning Calorimeter (DSC, TA Q20, New Castle, DE, USA) according to a modified method [23]. We prepared samples by weighing 2.5 mg of rice flour into an aluminum pan, adding 7.5 μL of distilled water, and hermetically sealing the pan. After equilibrating at 4 °C for 12 h, we heated the samples from 20 °C to 120 °C at 10 °C/min, using an empty pan as a reference. We obtained the onset (T_o_), peak (T_p_), and conclusion (T_c_) temperatures, as well as the gelatinization enthalpy (ΔH), directly from the DSC curve.

### 2.10. Rheological Properties Measurement

The rheological properties of the rice flour paste were characterized using a rheometer (Mars 60, HAAKE, Karlsruhe, Germany) equipped with a 40 mm diameter parallel-plate geometry and a 1 mm gap. The experimental procedure was adapted from a previous study with modifications [24]. The rheological properties of starch-based samples were analyzed using a strain-controlled rheometer equipped with a parallel plate geometry (e.g., 40 mm diameter, 1.0 mm gap), (Mars 60, HAAKE, Karlsruhe, Germany). A 2.00 g sample was prepared by gelatinizing 2% (*w*/*w*) of the total flour in 10 mL of distilled water in a 100 °C bath for 5 min. The remaining flour was incorporated into this hot paste, mixed thoroughly, and equilibrated at 25 °C for 30 min in a sealed container. Prior to testing, the linear viscoelastic region (LVER) was confirmed via an amplitude sweep, establishing a fixed strain of 0.1% for all measurements. A temperature sweep was then performed by heating from 25 °C to 100 °C at 3 °C/min, holding for 60 s, and cooling back to 25 °C at the same rate. A fixed frequency of 1 Hz was maintained, and evaporation was minimized with a solvent trap. The storage modulus (G′), loss modulus (G″), and loss tangent (tan δ) were recorded to evaluate the thermal gelation and viscoelastic behavior.

### 2.11. X-Ray Diffraction (XRD)

The X-ray diffraction (XRD) patterns of the rice flour samples were acquired using an X-ray diffractometer (D8 Advance, Bruker, Billerica, MA, USA) to determine their crystalline structure and crystallinity degree. The measurements were conducted with Cu Kα radiation (λ = 0.15406 nm) at 40 kV and 30 mA. The scanning range was set from 5° to 40° (2θ) with a scanning speed of 4°/min and a step size of 0.02°, following the previous method [25]. The degree of crystallinity was quantitatively calculated as the ratio of the integrated area of the sharp crystalline peaks to the total area under the diffraction spectrum.

### 2.12. Statistical Analysis

All experiments were conducted with at least three replicate measurements. Data are presented as the mean ± standard deviation. Statistical analysis was performed using SPSS software (SPSS, Statistics 29, Inc., Armonk, NY, USA). One-way analysis of variance (ANOVA) was applied, and Duncan’s multiple range test was used to determine significant differences among mean values at a 95% confidence level (*p* < 0.05).

## 3. Results and Discussion

### 3.1. Particle Size Distributions of Rice Flours

The particle size distributions of the five rice flour samples are summarized in Table 1. The parameters D10, D50, and D90 represent the equivalent diameters at which the cumulative volume reaches 10%, 50%, and 90%, respectively. A significant reduction in D10, D50, and D90 was observed with decreasing sieve mesh aperture (*p* < 0.05). D50 is commonly used to represent the median particle size; the values for the five rice flours followed the order: A (97.19 μm) > B (90.78 μm) > C (87.63 μm) > D (76.26 μm) > E (68.56 μm).

The damaged starch content was determined using an SDmatic damaged starch analyzer (Chopin Technologies). A higher iodine absorption value indicates a greater proportion of damaged starch. As D50 decreased from 97.19 μm to 68.56 μm, the iodine absorption value increased from 82.48% to 88.74%. This trend can be attributed to the fact that finer particles undergo more extensive mechanical and thermal damage during milling [26], resulting in a higher degree of starch granule disruption. During rice milling, starch granules are subjected to various mechanical stresses that break intact granules into smaller fragments [27], thereby increasing the damaged starch content. In comparison to intact granules, damaged starch exhibits higher water absorption capacity and enzymatic hydrolysis rates [28,29], underscoring the importance of quantifying starch damage. The extent of starch damage is influenced by several factors, including raw material properties, particle size, and milling conditions. Previous studies have reported a significant correlation between particle size distribution and damaged starch content, with finer particles generally associated with higher levels of damaged starch [30], which aligns with the findings of this study.

**Figure 1:**
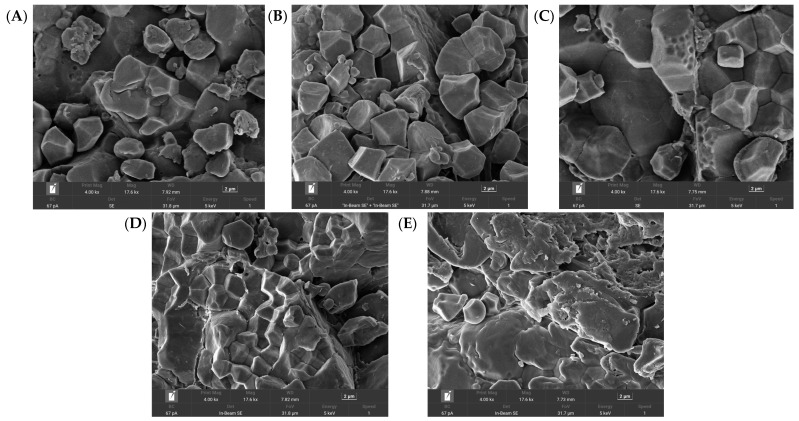
SEM images of five different grain sizes of rice flour at 4000× magnification. Note: Grain size of five types of rice flour: (**A**) (97.19 μm), (**B**) (90.78 μm), (**C**) (87.63 μm), (**D**) (76.26 μm) and (**E**) (68.56 μm).

Scanning electron microscopy (SEM) was used to further illustrate the effect of particle size distribution on the rice flour morphology (Figure 1). As the sieve pore size decreased, more fine particles and surface scratches became evident, consistent with the observed increase in damaged starch content. Particle size, influenced by processing methods, directly determines their functional properties. Finer particles (Figure 1E) have a larger specific surface area, leading to faster water absorption, enhanced swelling, and easier gelatinization, making them ideal for instant foods. However, reduced particle size also increases enzyme accessibility, potentially raising the digestion rate. Morphologically, smooth, intact granules (Figure 1A) indicate minimal processing damage, while fractured edges (Figure 1E) suggest intensive milling. This damage can increase free starch chains, adversely affecting product clarity and retrogradation. Consequently, ultra-fine flour (Figure 1E) is highly suitable for producing modified starch, as its high reactivity facilitates chemical reactions like esterification.

### 3.2. Chemical Compositions

The chemical compositions of rice flour across different particle size distributions are summarized in Table 1. Statistical analysis revealed no significant differences (*p* > 0.05) in the contents of protein, ash, total starch, or amylose with decreasing particle size. This result deviates from some previous reports, which indicated that protein, total starch, and amylose contents decrease with finer particles, whereas lipid content increases [31]. This discrepancy may be attributed to the inherent spatial heterogeneity in compositional distribution within the rice kernel, where protein concentration generally diminishes from the peripheral layers toward the endosperm core. In the current experimental setup, however, the milling of whole grains followed by sieving enabled each particle size fraction to retain a representative sampling of all anatomical regions, thereby preserving a consistent chemical profile across granulometric classes at a fixed flour extraction rate.

### 3.3. Color of Rice Flours

The whiteness of rice flour represents a critical determinant of visual appeal and overall acceptability in derived food products, with higher whiteness values generally correlating with enhanced consumer preference. As summarized in Table 2, color parameters (L*, a*, b*) exhibited significant variation (*p* < 0.05) across different particle size fractions. A progressive reduction in particle diameter was associated with a marked increase in L* (lightness), whereas the a* value remained largely stable with a subtle reddish hue. Conversely, the b* value (yellowness) demonstrated a consistent decline, resulting in a perceptible color shift from yellowish toward bluish tones and a pronounced improvement in overall whiteness, particularly when particle size was reduced to the range represented by sample B. These optical alterations may be ascribed to intensified particle fragmentation, wherein smaller particles—with median diameters decreasing from 97.19 μm to 68.56 μm—possess a greater specific surface area that enhances light scattering efficiency and thereby elevates perceived whiteness, consistent with principles of particulate light interaction reported in granular materials [27].

### 3.4. Hydration Properties Analysis

The hydration behavior of rice flour, governed primarily by the water-binding capacity of its starch and protein constituents, exerts a profound influence on the quality attributes of starch-based food products [27]. Specifically, water solubility (WS) and swelling power (SP) are closely associated with the breaking rate and cooking loss of fresh wet rice noodles, whereas the water absorption index (WAI) may determine the shear resistance of the final product. Similarly, the average particle size of rice flour significantly modulates its hydration performance, with finer particles offering a larger specific surface area that facilitates more extensive water contact and improved binding capacity [20].

As shown in Table 2, at 25 °C, no significant differences were observed in the WAI, WS, or SP of rice flour as the milling particle size decreased. However, when the temperature was raised to 100 °C, all three hydration parameters increased significantly compared to those at 25 °C. This can be attributed to the fact that rice flour remains largely insoluble in water below its gelatinization temperature. Upon heating, excess water disrupts the crystalline structure of starch, exposing more hydrogen bonds and enabling greater water absorption, particle swelling, and solubility. Furthermore, with decreasing particle size, WAI, WS, and SP demonstrated a consistent upward trend, a pattern likely driven by the combined effects of reduced granule dimensions, elevated damaged starch content, and diminished starch crystallinity, all of which collectively facilitate water penetration and the leaching of soluble components from fragmented granules.

### 3.5. Pasting Properties

The pasting behavior of starch is characterized by a hierarchical structural disassembly upon heating in aqueous medium, wherein hydrogen bonds within both crystalline and amorphous domains are progressively disrupted, thereby enabling the establishment of new intermolecular interactions between starch chains and water molecules that collectively drive granule swelling and subsequent gel network formation. This thermally induced transition amplifies structural disorder, diminishes crystalline integrity, and transforms the initial suspension into a cohesive viscous paste [24]. Within this framework, peak viscosity (PV) signifies the dynamic equilibrium between granule swelling and disintegration, serving as an indicator of the water-binding capacity of starch, whereas final viscosity (FV)—a defining parameter of pasting characteristics—reflects the gel-forming ability of the material upon cooling. Excessively high viscosity may impede the processability of rice noodles during molding, while insufficient viscosity often results in a poorly integrated structure and diminished cooking quality [6]. As shown in Table 3, both PV and FV of rice flour decreased significantly (*p* < 0.05) with decreasing particle size. However, beyond the threshold represented by sample C, further reduction to sample E exerted no statistically meaningful influence on PV, trough viscosity (TV), or FV. This apparent plateau may be attributed to the competing effects of particle size reduction and elevated damaged starch content—two factors known to exert opposing influences on paste viscosity, as finer particles typically enhance viscosity while starch damage suppresses it [14]. Under the concurrent action of these countervailing factors, the overall pasting profile remained largely unaltered in the finest fractions.

Breakdown viscosity (BD), calculated as the difference between PV and TV, serves as a measure of structural stability under thermal and shear stress, with higher values indicating lower stability. The observed reduction in BD with decreasing particle size implies enhanced thermal integrity in finer flours.

### 3.6. Thermal Properties

Thermal properties can measure the process of ordered structure destruction during starch pasting and reflect its thermodynamic changes. The onset temperature (T_o_) represents the temperature at which the crystal structure begins to break, reflecting the stability of the crystal structure. The peak temperature (T_p_) represents the temperature at which the starch absorbs the fastest heat during gelatinization, and the termination temperature (T_c_) is the temperature at which the starch structure is destroyed. The gelatinization enthalpy (ΔH) indicates the combination of the heat absorbed during the destruction of the ordered structure of rice flour during gelatinization and the heat released during particle expansion, that is, the energy required for crystal melting. The smaller the enthalpy change value, the lower the degree of ordering of starch crystals.

The thermal properties are shown in Table 3. There are significant differences between the samples. With the decrease in particle size, T_o_, T_p_ and T_c_ of rice flour decrease slightly. This may be mainly related to the damaged starch content of rice flour. Relevant studies have shown that T_o_, T_p_, T_c_ and ΔH of rice flour are negatively correlated with the damaged starch content, mainly due to the higher water absorption rate with the damaged starch, so it is more conducive to the gelatinization of rice flour. In this study, ΔH decreased from 11.12 J/g to 8.29 J/g with reduced particle size, indicating a diminished energy requirement for pasting that arises from alterations in the starch crystalline architecture. The crystalline framework in rice starch consists predominantly of amylopectin clusters stabilized by inter-chain hydrogen bonding, with minor contributions from amylose double helices. Lower crystallinity promotes the leaching of amylose during heating, accelerates the disintegration of crystalline domains, and reduces the overall energy needed for melting. These findings align with earlier reports that higher crystallinity correlates with increased initial gelatinization temperatures and greater enthalpy values [24], and are further consistent with the reduced crystallinity observed in complementary structural analyses.

### 3.7. Rheological Properties

**Figure 2:**
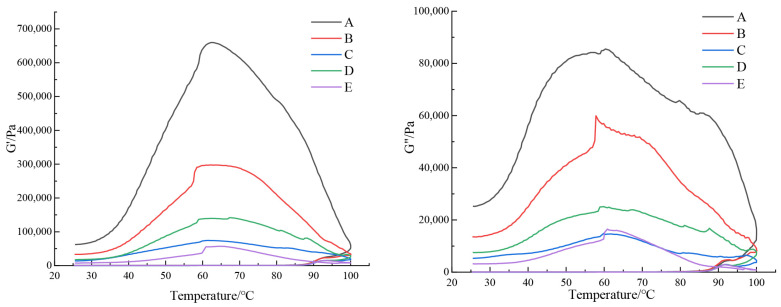
Dynamic storage modulus (G′) and loss modulus (G″) changes in rice flour with different particle size distributions. Grain size of five types of rice flour: A (97.19 μm), B (90.78 μm), C (87.63 μm), D (76.26 μm) and E (68.56 μm).

The rheological behavior of gel systems, which characterizes the evolution of their elastic and viscous properties under applied stress, provides critical insights for evaluating and predicting the performance of food matrices. The storage modulus (G′) indicates the elasticity of the sample, which refers to the energy recovered by deformation after a vibration period, and can represent the solid behavior of the sample. The loss modulus (G″) indicates the viscous nature of the sample, which means that the energy consumed after a period of deformation can represent the fluid properties of the sample. The loss tangent Tanδ is the ratio of G″ to G′, which describes the viscosity and elastic strength of the sample. The higher the value, the greater the viscosity ratio of the sample. The rheological properties provided by the starch matrix are the key factors affecting the processability of rice flour and the quality of final products [21]. The curves of G′ and G″ with temperature during heating and cooling are shown in Figure 2.

Rice flours with distinct particle size distributions exhibited markedly different rheological profiles during a temperature sweep from 25 °C to 100 °C. As can be seen in Figure 2, both G′ and G″ remained relatively stable in the initial heating phase before increasing sharply near 85 °C, a transition attributable to the disruption of hydrogen bonds, granule dissolution, and leaching of amylose molecules from swollen starch granules. This melting of crystalline domains led to an initial rise in system viscosity, followed by the extension of amylopectin chains and their entanglement with leached amylose, collectively promoting the formation of a continuous three-dimensional gel network that drove the rapid increase in both G′ and G″. In flours with larger particle sizes (fractions A–D), G′ and G″ displayed a temporary decrease or plateau around 92 °C before resuming an upward trajectory, likely due to their more intact granule architecture, lower damaged starch content, and higher crystallinity, which delayed full network development. In contrast, the finest fraction (E) exhibited a decline in both moduli beyond this temperature, suggesting that excessive heating melted the remaining crystalline regions, softened the granules, and induced shear-induced breakdown of the gel network, thereby reducing overall viscoelasticity.

During cooling from 100 to 25 °C, G′ and G″ initially increased but eventually decreased with temperature, a pattern consistent with starch retrogradation and molecular reorganization. At the same time, with the decrease in the particle size of the rice flour, it can be seen that the final G′ and G″ show a decreasing trend, which indicates that its viscoelasticity decreases. The damaged starch content of rice flour with a small range of particle size distribution is high, and a poor ability to form an ordered spatial network structure between molecular 90.78 μm ns throughout the testing process, thus its viscoelasticity decreases. Across all heating and cooling stages, G′ consistently exceeded G″ (tan δ < 1), indicating dominant elastic behavior and solid-like character in the rice flour gels, with particle size exerting a clear influence on the structural integrity and viscoelastic performance of the resulting network [31].

### 3.8. X-Ray

Starch granule is a kind of semi-crystalline structure, which is mainly composed of crystalline region and non-crystalline region. The intermolecular structure in the crystalline region is relatively dense, and the peak diffraction characteristics will appear in the X-ray diffraction spectra. Therefore, the diffraction effect of X-ray in crystal material can be used to analyze the material structure. Starch with different crystallinity and crystalline forms will show different properties in life. Usually, the crystal types are divided into three types: A-type, B-type and C-type. A-type starch has strong diffraction peaks at 15, 17, 18 and 23°. The strong diffraction peak angles of B-type starch are 5, 8, 15, 17 and 20°, and the strong diffraction peak angles of C-type starch are 5.5, 15, 17 and 23 [25].

**Figure 3:**
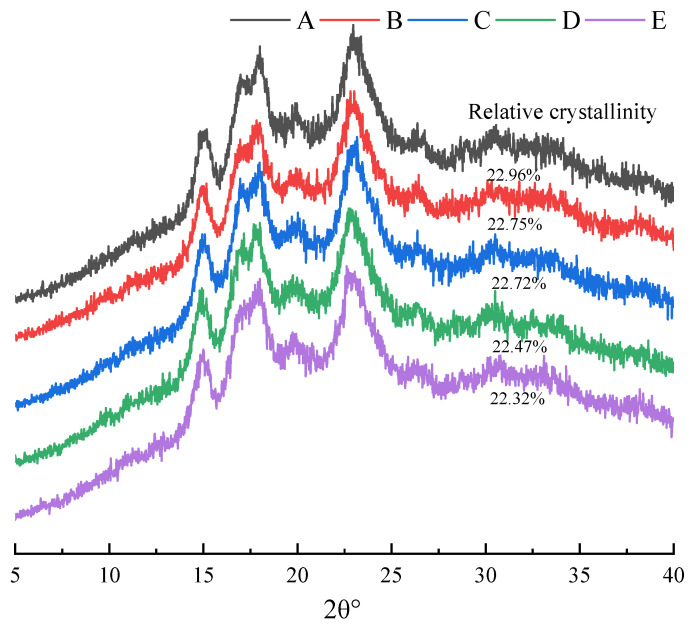
X-ray of rice flour with different particle size distributions. Grain size of five types of rice flour: A (97.19 μm), B (90.78 μm), C (87.63 μm), D (76.26 μm) and E (68.56 μm).

As shown in Figure 3, XRD profiles of rice flours with varying particle distributions displayed consistent diffraction angles across all samples, featuring characteristic peaks at 15°, 17°, 18°, and 23° that confirm the typical A-type polymorphic structure consistent with previous reports [25], This consistency indicates that particle size distribution does not alter the fundamental crystalline architecture of starch granules. Relative crystallinity, defined as the ratio of crystalline diffraction area to total diffraction area, represents a crucial parameter determining functional performance in industrial applications. Quantitative analysis revealed distinct crystallinity values across different particle size fractions, progressively decreasing from 22.96% to 21.12% with diminishing particle size. This reduction in peak intensity suggests mechanical degradation of amylopectin crystalline clusters during comminution, wherein the grinding process disrupts long-range molecular order in crystalline regions, facilitating transformation to amorphous states. The observed crystallinity decrease thus results from combined effects of crystalline domain disintegration and expansion of amorphous content [4,26], providing mechanistic insight into how particle size reduction modifies starch functionality through structural reorganization.

## 4. Conclusions

Particle size distribution serves as a critical determinant influencing the physicochemical behavior of rice flour, despite exerting no statistically significant effect on major chemical constituents such as total protein and amylose content. With progressive reduction in particle size, a marked increase in whiteness and damaged starch content was observed, indicative of intensified mechanical damage during milling. Pasting parameters—including peak viscosity, final viscosity, breakdown, and setback—displayed a consistent decline, plateauing below the threshold of 124 μm. Concurrently, thermal and rheological properties revealed a downward trend in gelatinization temperatures, enthalpy change, and dynamic moduli, suggesting attenuated structural integrity and weakened gel network formation in finer flours. Notably, all samples maintained an A-type crystalline pattern, implying that crystalline polymorphism remains unaltered by granulometry. Furthermore, the elevated water absorption index, water solubility, and swelling power at 100 °C underscore the enhanced hydration capacity of finer particles under high-temperature conditions. However, this study did not investigate the effects of different particle sizes of rice flour on the quality of other rice products, such as rice cakes and rice tofu. Collectively, these findings elucidate the structure–function interplay governed by particle size in rice flour, providing a theoretical basis for its tailored application in starch-based food systems and process optimization.

## Figures and Tables

**Table 1:** Particle size distribution and chemical composition of rice flour with different particle size distributions.

Rice Sample	D_10_ (μm)	D_50_ (μm)	D_90_ (μm)	Fat (%)	Ash (%)	Protein (%)	Total Starch (%)	Amylose (%)	AI (%)
A	47.40 ± 0.05 ^a^	97.19 ± 0.69 ^a^	166.34 ± 0.78 ^a^	0.60 ± 0.00 ^a^	0.62 ± 0.01 ^a^	9.03 ± 0.13 ^a^	80.87 ± 0.10 ^a^	21.47 ± 0.01 ^a^	82.48 ± 0.37 ^e^
B	45.44 ± 0.28 ^b^	90.78 ± 0.59 ^b^	154.18 ± 0.92 ^b^	0.62 ± 0.04 ^a^	0.59 ± 0.00 ^a^	8.97 ± 0.04 ^a^	80.49 ± 0.58 ^a^	21.14 ± 0.17 ^a^	83.68 ± 0.13 ^d^
C	44.61 ± 0.22 ^b^	87.63 ± 0.49 ^c^	149.37 ± 0.98 ^c^	0.60 ± 0.030 ^a^	0.61 ± 0.01 ^a^	8.95 ± 0.01 ^a^	80.09 ± 0.32 ^a^	21.75 ± 0.01 ^a^	85.23 ± 0.15 ^c^
D	37.63 ± 1.35 ^c^	76.26 ± 1.43 ^d^	131.5 ± 1.61 ^d^	0.61 ± 0.01 ^a^	0.61 ± 0.05 ^a^	8.91 ± 0.05 ^a^	80.76 ± 0.36 ^a^	21.86 ± 0.54 ^a^	87.47 ± 0.03 ^b^
E	35.01 ± 0.13 ^d^	68.56 ± 0.13 ^e^	118.65 ± 1.83 ^e^	0.63 ± 0.02 ^a^	0.65 ± 0.01 ^a^	8.87 ± 0.05 ^a^	80.31 ± 0.43 ^a^	21.81 ± 0.4 ^a^	88.74 ± 0.06 ^a^

Note: AI, Absorption Index. Different letters in a column indicate significant differences (*p* < 0.05), while the same letter indicates no significant difference (*p* > 0.05). A (97.19 μm), B (90.78 μm), C (87.63 μm), D (76.26 μm) and E (68.56 μm).

**Table 2:** Hydration properties and color of rice flour with different particle size distributions.

Rice Sample	WAI (g/g)		WS (%)		SP (g/g)		L*	a*	b*	Whiteness
	25 °C	100 °C	25 °C	100 °C	25 °C	100 °C				
A	2.07 ± 0.15 ^a^	9.82 ± 0.10 ^c^	3.44 ± 0.16 ^a^	13.65 ± 0.60 ^b^	2.16 ± 0.17 ^a^	11.37 ± 0.04 ^d^	90.48 ± 0.06 ^d^	3.24 ± 0.01 ^b^	0.09 ± 0.04 ^a^	89.94 ± 0.05 ^d^
B	2.19 ± 0.08 ^a^	10.60 ± 0.07 ^b^	3.57 ± 0.17 ^a^	14.86 ± 1.28 ^ab^	2.27 ± 0.09 ^a^	12.45 ± 0.11 ^c^	90.77 ± 0.02 ^c^	3.27 ± 0.01 ^a^	−0.29 ± 0.01 ^b^	90.20 ± 0.02 ^c^
C	2.23 ± 0.03 ^a^	10.89 ± 0.03 ^a^	3.74 ± 0.34 ^a^	14.24 ± 0.13 ^ab^	2.31 ± 0.04 ^a^	12.70 ± 0.06 ^bc^	90.82 ± 0.12 ^bc^	3.24 ± 0.01 ^b^	−0.36 ± 0.06 ^b^	90.26 ± 0.11 ^bc^
D	2.36 ± 0.06 ^a^	11.00 ± 0.19 ^a^	3.84 ± 0.23 ^a^	15.64 ± 0.26 ^a^	2.44 ± 0.06 ^a^	13.04 ± 0.19 ^a^	90.96 ± 0.04 ^ab^	3.24 ± 0.00 ^b^	−0.61 ± 0.03 ^c^	90.37 ± 0.03 ^ab^
E	2.33 ± 0.26 ^a^	10.96 ± 0.04 ^a^	4.07 ± 0.73 ^a^	14.65 ± 0.25 ^ab^	2.43 ± 0.28 ^a^	12.84 ± 0.01 ^ab^	91.12 ± 0.04 ^a^	3.25 ± 0.01 ^b^	−0.63 ± 0.01 ^c^	90.52 ± 0.03 ^a^

Note: WAI, the water absorption index; WS, water solubility index; SP, swelling power. Different letters in a column indicate significant differences (*p* < 0.05), while the same letter indicates no significant difference (*p* > 0.05). A (97.19 μm), B (90.78 μm), C (87.63 μm), D (76.26 μm) and E (68.56 μm).

**Table 3:** RVA pasting viscosity and thermal properties of rice flour with different particle size distributions.

Rice Sample	PV (cP)	TV (cP)	BV (cP)	FV (cP)	SV (cP)	PT (°C)	T_o_ (°C)	T_P_ (°C)	T_C_ (°C)	ΔH (J/g)
A	3768.50 ± 23.33 ^a^	2858.51 ± 30.41 ^a^	910.00 ± 7.07 ^a^	5785.52 ± 2.12 ^a^	2927.01 ± 32.53 ^a^	81.53 ± 0.67 ^a^	73.62 ± 0.08 ^a^	77.90 ± 0.18 ^a^	86.58 ± 0.09 ^a^	11.12 ± 0.06 ^a^
B	3625.52 ± 43.13 ^b^	2843.30 ± 206.48 ^a^	782.53 ± 163.34 ^a^	5600.51 ± 149.20 ^a^	2757.51 ± 57.28 ^b^	81.10 ± 0.00 ^ab^	73.39 ± 0.21 ^ab^	77.79 ± 0.28 ^ab^	86.36 ± 0.13 ^a^	10.91 ± 0.08 ^a^
C	3528.0 ± 63.64 ^bc^	2727.03 ± 0.00 ^a^	801.06 ± 63.64 ^ab^	4953.53 ± 9.19 ^b^	2216.52 ± 23.33 ^c^	79.93 ± 0.53 ^b^	73.09 ± 0.04 ^b^	77.40 ± 0.01 ^b^	86.36 ± 0.34 ^a^	10.49 ± 0.05 ^b^
D	3482.05 ± 0.00 ^c^	2806.51 ± 68.59 ^a^	675.51 ± 68.59 ^b^	4831.50 ± 2.12 ^b^	2025.02 ± 70.71 ^d^	80.30 ± 0.07 ^b^	73.16 ± 0.04 ^b^	77.37 ± 0.13 ^b^	86.34 ± 0.11 ^a^	9.15 ± 0.13 ^c^
E	3531.51 ± 60.10 ^bc^	2867.02 ± 38.18 ^a^	664.51 ± 21.92 ^b^	4925.51 ± 86.97 ^b^	2058.50 ± 48.79 ^d^	79.90 ± 0.57 ^b^	73.18 ± 0.24 ^b^	77.50 ± 0.03 ^ab^	85.46 ± 0.25 ^b^	8.29 ± 0.23 ^d^

Note: Different letters in a column indicate significant differences (*p* < 0.05), while the same letter indicates no significant difference (*p* > 0.05). (Pasting Temperature, PT), (Temperature Onset, T_o_), (Peak Viscosity, PV), (Temperature Peak, T_P_), (Trough Viscosity, TV), (Breakdown Viscosity, BV), (Final Viscosity, FV), (Setback Viscosity, SV), (Temperature Conclusion, T_C_).

## Data Availability

The original contributions presented in this study are included in the article. Further inquiries can be directed to the corresponding authors.
